# Pneumomediastinum, Pneumopericardium, and Pneumothorax: An Uncommon Triad Induced by Cocaine Use

**DOI:** 10.7759/cureus.99947

**Published:** 2025-12-23

**Authors:** Md Hasanur Rahman, MD Hassan Sazu, Bianca Afroz Liya, Geerthana Satchithananthan, Md Rafsan Janee, Amreen Sarker

**Affiliations:** 1 General Internal Medicine, Luton and Dunstable University Hospital, Luton, GBR; 2 General Practice, Choudhury Medical Care PC, Brooklyn, USA; 3 General Internal Medicine, Sher-E-Bangla Medical College, Barishal, BGD

**Keywords:** cocaine-induced barotrauma, cocaine inhalation, cocaine inhalation complications, cocaine intoxication, cocaine lung injury, cocaine toxicity, pneumothorax (ptx), simple pneumopericardium, spontaneous pneumomediastinum (spm)

## Abstract

We present a rare case of a clinically stable 24-year-old male patient who developed acute central chest pain shortly after intranasal cocaine use. Initial chest imaging revealed pneumomediastinum, a small apical pneumothorax, and pneumopericardium-three uncommon yet potentially serious findings. Remarkably, the patient was hemodynamically stable with unremarkable clinical examination and vital signs. High-resolution computed tomography (CT) of the thorax and abdomen with oral and intravenous contrast effectively excluded esophageal rupture, a critical differential diagnosis requiring surgical intervention. The patient was successfully managed with conservative measures including observation and supportive care. Serial imaging demonstrated progressive improvement, with complete radiological resolution confirmed at follow-up. This case underscores the need for clinicians to consider recreational drug use as a cause of thoracic barotrauma, utilize imaging judiciously to exclude life-threatening conditions, and recognize that conservative management may be appropriate in carefully selected, clinically stable patients.

## Introduction

Pneumomediastinum, pneumothorax, and pneumopericardium involve the abnormal presence of air in the mediastinum, pleural space, and pericardial sac, respectively. These conditions may arise from trauma, pulmonary disease, or iatrogenic causes such as medical procedures [1,2]. Spontaneous pneumomediastinum (SPM) and pneumothorax are known but uncommon complications of cocaine inhalation, believed to result from alveolar rupture due to increased intrathoracic pressures [3-7]. Pneumopericardium is even more rare [8,9]. Differentiating between benign barotrauma and life-threatening causes such as esophageal rupture is critical for safe and effective management [10-13].

## Case presentation

A 24-year-old male ex-smoker presented to the emergency department with central chest pain following recreational cocaine inhalation. He denied vomiting, breathlessness, hematemesis, or any history of trauma. He had no prior history of respiratory, cardiovascular, or gastrointestinal disease and no relevant family history.

On presentation, he was afebrile, with a heart rate of 82 beats per minute (bpm), a respiratory rate of 18 breaths per minute, SpO_2_ of 96% on room air, and blood pressure of 105/67 mmHg. Physical examination, including a cardiopulmonary exam, was unremarkable.

Laboratory studies demonstrated a slight elevation of C-reactive protein (CRP), with no other significant abnormalities (Table 1). His admission electrocardiogram (ECG) confirmed a baseline of sinus rhythm at 62 bpm.

**Table 1:** Laboratory results INR: international normalized ratio

Parameter	Result	Reference range
Sodium	139 mmol/L	133-146 mmol/L
Potassium	4.2 mmol/L	3.5-5.3 mmol/L
C-reactive protein	10 mg/L	0-4.9 mg/L
Cardiac troponin T	7.9 ng/L	0-14 ng/L
White blood cells	8.5 × 10^9^/L	4-11 × 10^9^/L
Neutrophil count	4.39 × 10^9^/L	2-7 × 10^9^/L
Lymphocyte count	2.98 × 10^9^/L	1-3 × 10^9^/L
Hemoglobin	142 g/L	130-165 g/L
Hematocrit	0.41 L/L	0.41-0.51 L/L
Platelet count	264 × 10^9^/L	150-450 × 10^9^/L
Alkaline phosphatase	55 U/L	30-130 U/L
Alanine aminotransferase	15 U/L	0-40 U/L
Creatinine	68 µmol/L	62-106 µmol/L
Prothrombin time	12.1 seconds	9-14 seconds
INR	1.1	0.8-1.2

**Figure 1:**
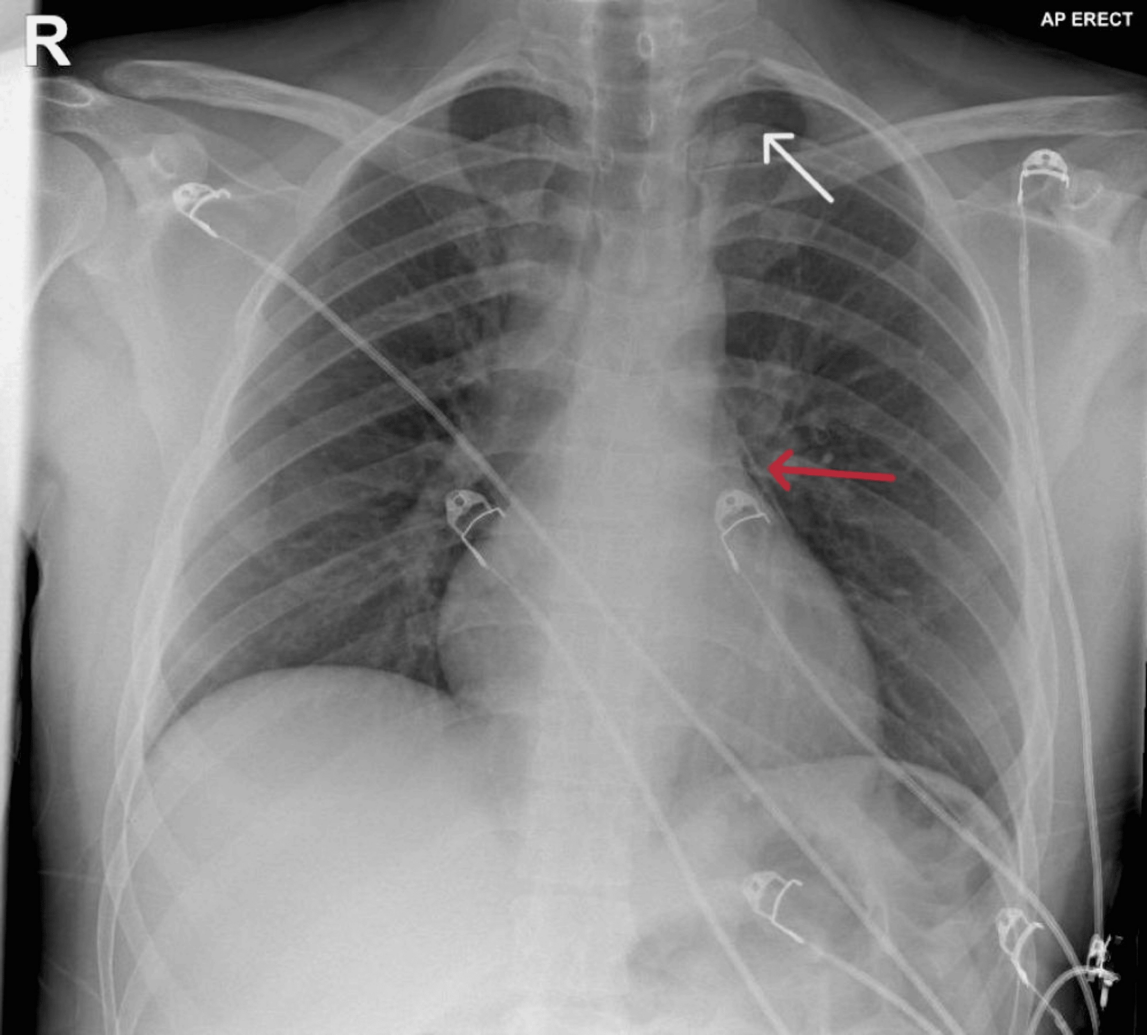
Chest X-ray on admission showing tiny left apical pneumothorax (<1 cm in width) (white arrow) and pneumomediastinum at the left cardiac border (red arrow)

In terms of imaging, a chest X-ray was performed, which demonstrated a tiny left apical pneumothorax (<1 cm in width) and pneumomediastinum at the left cardiac border (Figure 1).

**Figure 2:**
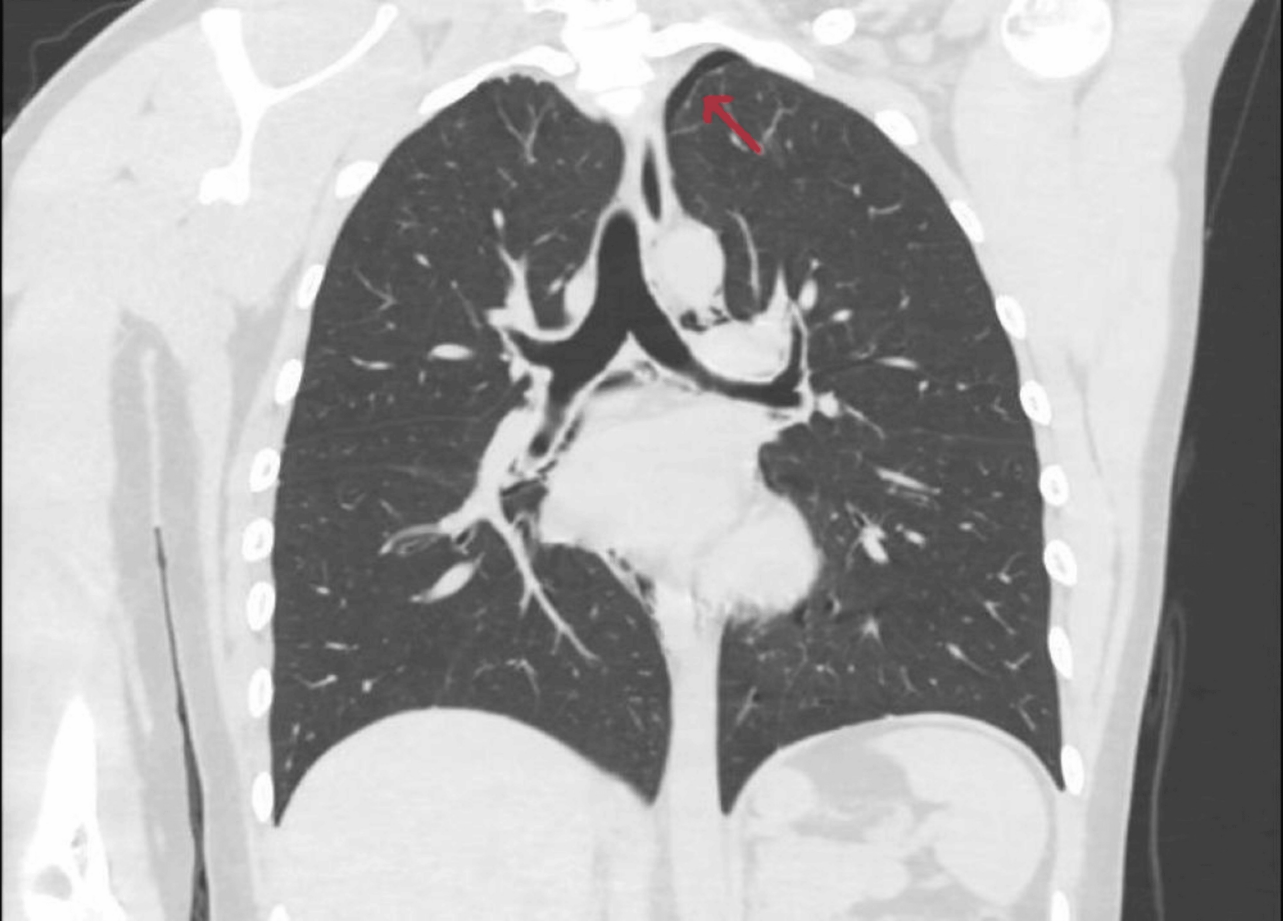
Computed tomography (CT) scan showing a small left pneumothorax (red arrow)

**Figure 3:**
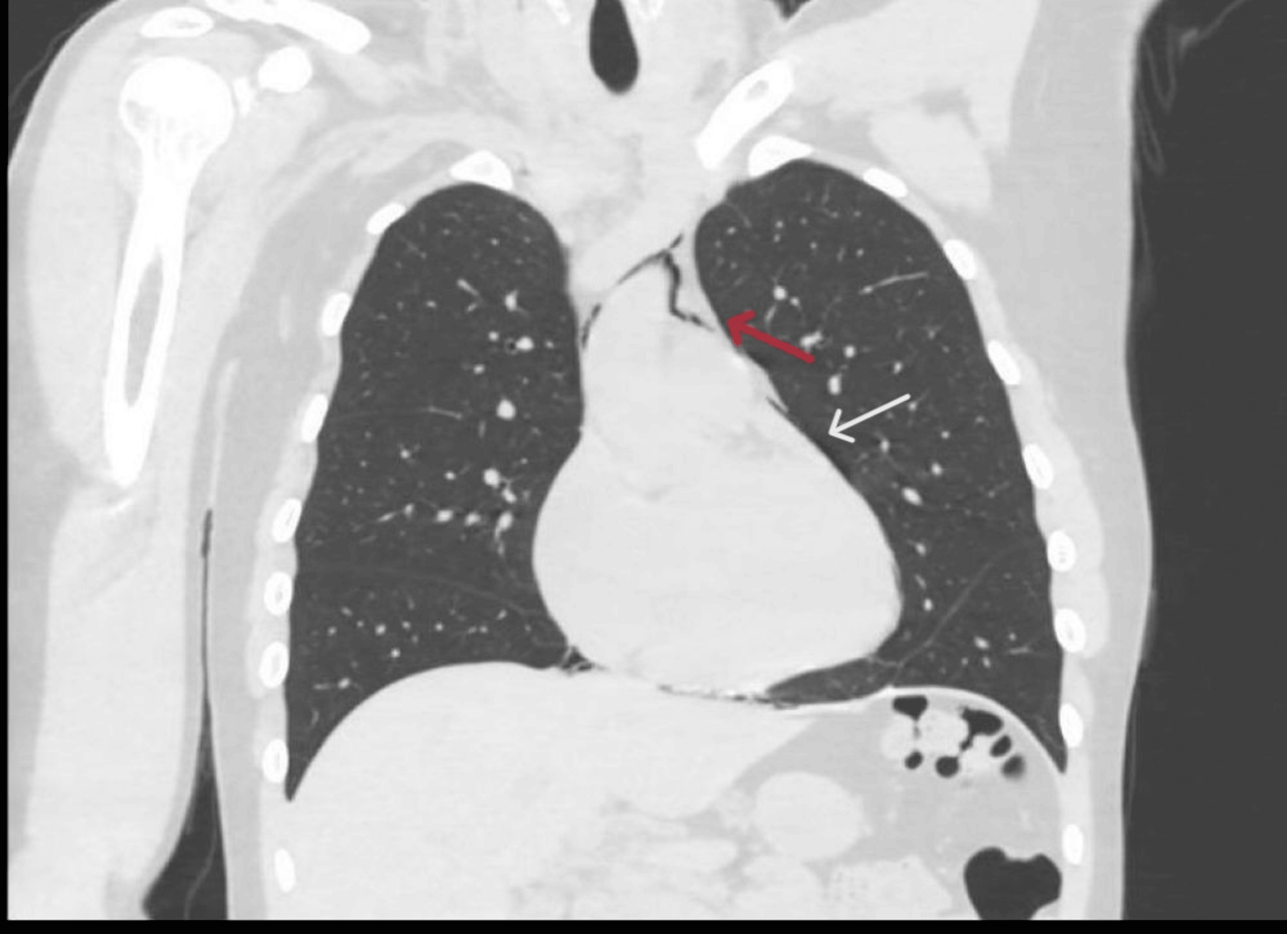
Computed tomography (CT) scan showing pneumomediastinum (red arrow) and pneumopericardium (white arrow)

A computed tomography (CT) angiogram aorta scan was performed, which showed a small left pneumothorax (Figure 2), pneumomediastinum, and pneumopericardium (Figure 3).

The cardiothoracic team was notified, and they deemed that these findings may be secondary to esophageal rupture, also known as Boerhaave’s syndrome. They advised a CT thorax abdomen with oral and intravenous (IV) contrast; the scan excluded esophageal perforation.

**Figure 4:**
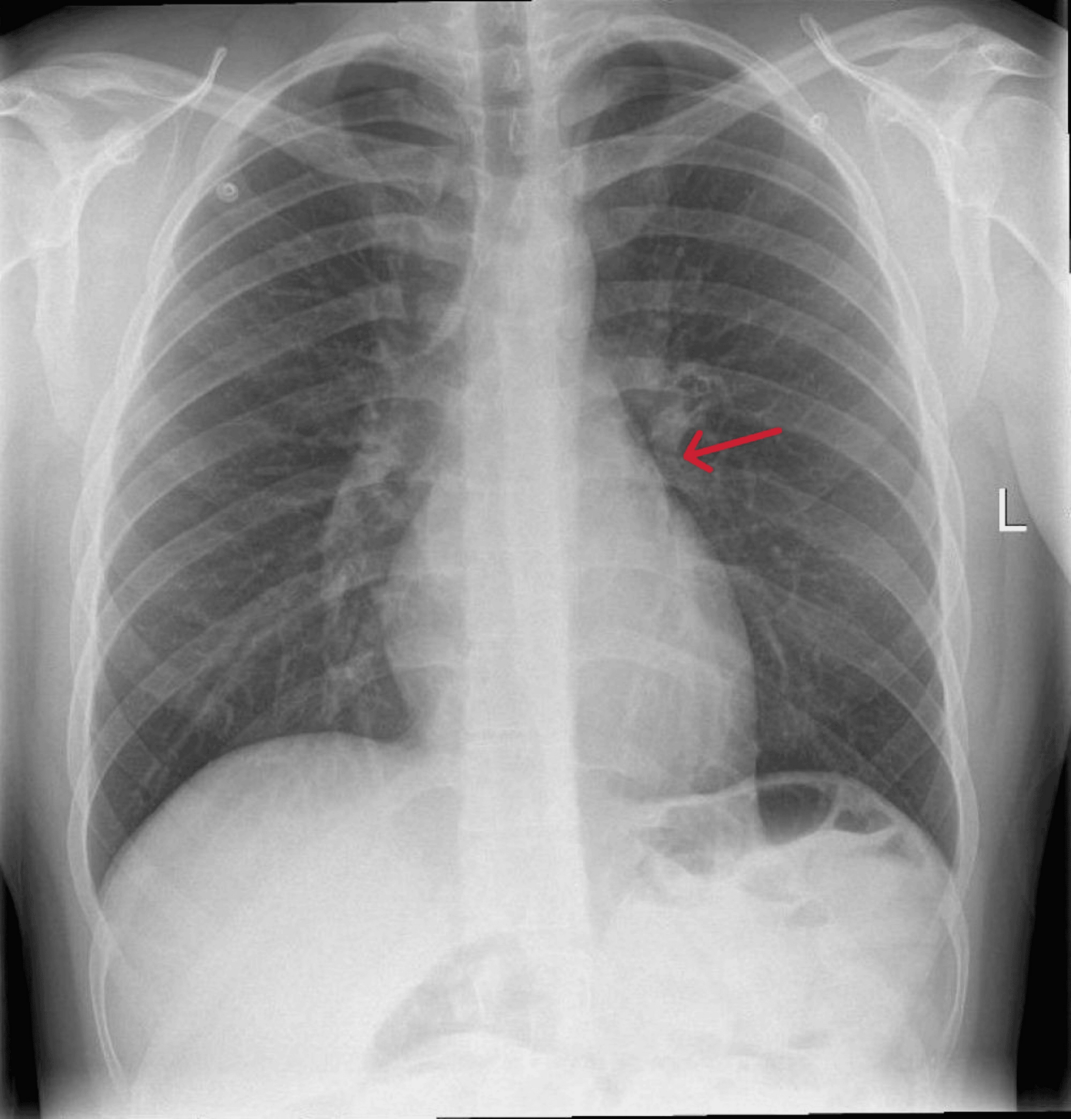
On day 3 of admission, chest X-ray showing resolving pneumomediastinum (red arrow) and no overt pneumothorax

He was initially treated with IV antibiotics. However, in the light of unremarkable inflammatory markers, that was stopped. On admission, he was reviewed by the respiratory team and attributed the findings to barotrauma secondary to cocaine inhalation. As the pneumothorax was small, no chest drain was indicated. Then, he was treated conservatively. On the third day of admission, he had a repeat chest X-ray, which demonstrated resolving pneumomediastinum and no overt pneumothorax (Figure 4). Then, he was discharged with outpatient respiratory clinic follow-up in six weeks and safety-netted to seek medical attention in the case of worsening breathlessness and chest pain.

**Figure 5:**
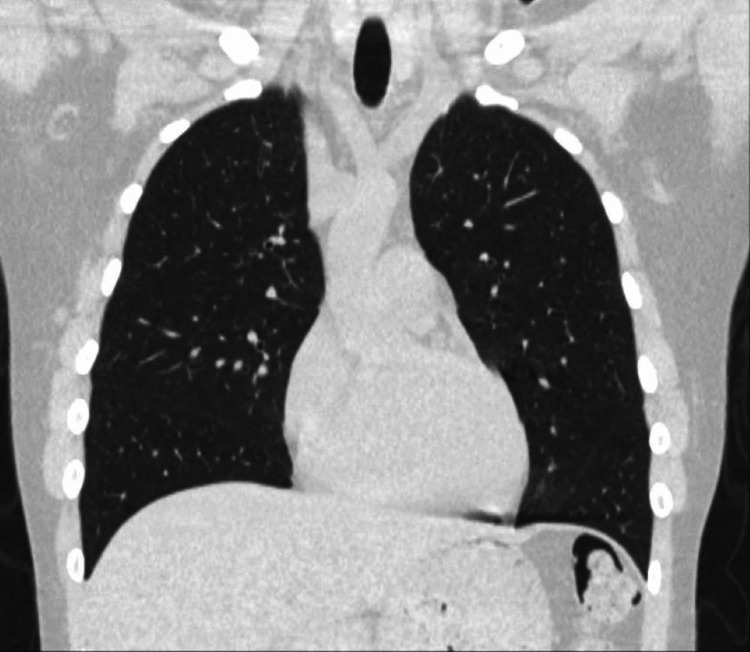
After 6 weeks, high-resolution chest computed tomography (CT) showing complete resolution of the pneumomediastinum, pneumopericardium, and left apical pneumothorax

After six weeks of respiratory clinic follow-up, high-resolution chest CT showed complete resolution of the pneumomediastinum, pneumopericardium, and left apical pneumothorax (Figure 5).

## Discussion

Pneumomediastinum is a rare clinical condition that can be associated with substance abuse, particularly inhaled drugs such as cocaine. It is more commonly seen secondary to iatrogenic causes, including endoscopic procedures and mechanical ventilation, or due to factors such as forceful vomiting, intense physical exertion, or underlying respiratory pathology like asthma or emphysema [1-3]. When no clear cause such as intrathoracic visceral perforation is identified, the condition is classified as SPM. SPM was first described by Louis Bourgeois in 1617 in association with straining during childbirth. When accompanied by subcutaneous emphysema, the presentation is referred to as Hamman’s syndrome, a rare entity named after Louis Virgil Hamman, who formally characterized it in 1939.

Cocaine inhalation can lead to barotrauma through the Macklin effect, a pathophysiological mechanism first described by Macklin in 1944 [4]. It involves alveolar rupture caused by a sudden increase in intra-alveolar pressure, with air dissecting along the bronchovascular sheaths into the mediastinum and potentially the pericardium. An alternative explanation suggests the direct toxic effect of cocaine on lung tissue, leading to alveolar rupture [5-8].

Importantly, esophageal rupture must be excluded as it presents with similar radiological findings but carries a high mortality risk. In this case, oral contrast CT imaging confirmed an intact esophagus, justifying conservative management.

There are currently no established guidelines for the management of cocaine-induced pneumomediastinum; however, most reported cases have been treated conservatively with favorable outcomes and radiological resolution on outpatient follow-up. Surgical intervention is typically reserved for cases complicated by esophageal perforation or other underlying structural injuries [5,6,9-13].

## Conclusions

Pneumomediastinum is a rare complication of cocaine misuse, most commonly associated with nasal insufflation. The proposed mechanism involves a Valsalva-like maneuver intended to enhance drug absorption, which increases intra-alveolar pressure and leads to alveolar rupture. Patients typically present with chest pain and dyspnea. This case highlights the potential for extensive thoracic air dissection secondary to cocaine use in a clinically stable patient. Although chest radiography can reveal mediastinal air, CT offers greater diagnostic accuracy and is essential for detailed evaluation and confirmation. Accurate imaging, collaborative multidisciplinary assessment, and vigilant clinical monitoring facilitated successful conservative management without the need for surgical intervention.

## References

[1] Singh D, Kumar S, Stead TS, Ganti L (2018). Spontaneous mediastinal emphysema. Cureus.

[2] Hawkins L, Khalid MA, Barton A (2022). Pneumomediastinum and subcutaneous emphysema post cocaine and amphetamine insufflation. JRSM Open.

[3] Jaensch S, Hwang S, Kuo TS (2019). Spontaneous pneumomediastinum and subcutaneous emphysema following cocaine inhalation and ecstasy ingestion. Case Rep Otolaryngol.

[4] Nelson LS (2025). Cocaine: acute intoxication. UpToDate.

[5] Molina G, Contreras R, Coombes K, Barbery M (2023). Pneumomediastinum as a complication of cocaine insufflation. Cureus.

[6] Jaramillo Escobar SL, Giraldo Campillo D, Reyes Romero K, Neira Rincón MA, Zuluaga M, Ardila CM (2024). Spontaneous pneumomediastinum and subcutaneous emphysema: a rare complication associated with cocaine consumption. Cureus.

[7] Macrae C, Brown C, Aiken C, Jamdar R (2019). Pneumomediastinum as a complication of cocaine abuse. Clin Med (Lond).

[8] Burns J, Roby A, Jaconelli T (2020). Pneumomediastinum, subcutaneous emphysema and pneumorrhachis following cocaine insufflation: a case report. Acute Med.

[9] Chudasama K, Seenath M, Gourevitch D (2010). Pneumomediastinum after cocaine use: an unusual aetiology. J Surg Case Rep.

[10] Dadhwal R, Bulathsinghala CP, Choudhry I, Taweesedt PT, Surani S (2021). Cocaine-induced bronchial laceration: a rare incidence. Cureus.

[11] Alnas M, Altayeh A, Zaman M (2010). Clinical course and outcome of cocaine-induced pneumomediastinum. Am J Med Sci.

[12] Abdeen AM, Lim O, Crow S, Watson V (2025). Chest pain beyond the heart: the role of cocaine in pneumomediastinum. AIM Clinical Cases.

[13] Chua JJ, Lee D, Buddhdev A (2024). Spontaneous pneumomediastinum as the result of marijuana use. AIM Clinical Cases.

